# Oxygen/phosphorus co-doped porous carbon from cicada slough as high-performance electrode material for supercapacitors

**DOI:** 10.1038/s41598-019-41769-y

**Published:** 2019-04-01

**Authors:** Bingwei Chen, Wenzhuo Wu, Chunyang Li, Yanfang Wang, Yi Zhang, Lijun Fu, Yusong Zhu, Lixin Zhang, Yuping Wu

**Affiliations:** 1 State Key Laboratory of Materials-oriented Chemical Engineering & School of Energy Science and Engineering, Nanjing Tech University, Nanjing, 211816 China; 2 New Energy and Materials Laboratory (NEML), Department of Chemistry and Shanghai Key Laboratory of Molecular Catalysis and Innovative Materials, Fudan University, Shanghai, 200433 China; 3 Guanghua Cambridge International School, Shanghai, 201315 China

## Abstract

Synthesizing high-performance electrode materials plays a vital role in fabricating advanced supercapacitors. Heteroatom doping has been proved to be effective in enhancing the electrochemical properties of carbon-based electrodes. Herein, we report an O, P co-doped porous carbon (PC) originated from waste biomaterials, cicada sloughs. The PC possesses meso-/microporous structure with a large specific surface area (1945 m^2^·g^−1^) and a high O, P co-doping ratio of 18 wt.%. These superior factors together enable it to deliver high specific capacitance (295 F·g^−1^ in 6 M KOH and 291 F·g^−1^ in 1 M H_2_SO_4_), good cycling stability (100% capacitance retention after 10000 cycles in 1 M H_2_SO_4_) and rate performance. Therefore, from the respects of environment friendliness and cost effectivity, obtaining heteroatom doped carbons from the nature might be better compared to pyrolyzing heteroatom-containing chemicals.

## Introduction

Recently, due to the climate change and depletion of fossil fuels, developing high-performance, low cost and environmentally-friendly energy storage and conversion systems is of great significance^1,2^. Because supercapacitors could deliver high power densities and long cycle lives (>10^5^), they are regarded as promising devices bridging the power/energy gap between dielectric capacitors and traditional batteries^3–5^. Depending on the charge storage mechanism, supercapacitors can be classified into pseudo-capacitors and electrical double layer capacitors (EDLCs)^3^. Pseudo-capacitors store charges by fast and reversible redox reactions between the electrolyte and electrode materials, which allow them to deliver high energy densities comparable to those of batteries^6^. However, hindered by drawbacks such as poor cycling stability and low conductivity, they are not ideal for practical applications yet. As for EDLCs, they store energy through pure electrostatic charge accumulation occurring at the electrode/electrolyte interfaces, which means that they could store and release charges fast^7^. Because the capacitances and rate properties are dependent on specific surface area (SSA), pore size, electrical conductivity and wettability, porous carbons (PC) including activated carbons (AC), ordered mesoporous carbons (OMC), carbon nanotubes (CNT) and graphene are used exclusively in EDLCs^8–10^.

Among them, ACs have drawn wide attention owing to their controllable pore size, high SSA (>1000 m^2^·g^−1^), large pore volume (>0.5 cm^3^·g^−1^), excellent thermal stability and low cost^11^. Traditional ACs, with SSA of more than 3000 m^2^·g^−1^, always show undesirable capacitances for EDLCs because of the sluggish ion diffusion in narrow micropores. Also, their poor electronic conductivity and long ion pathway induce high internal resistance and inferior rate performance.

In the past years, fabricating hierarchical and surface functionalized structures has been proved to be effective in improving their properties. For example, unlike traditional activation using KOH, ZnCl_2_ or H_3_PO_4_ as the chemical activating agents. Xu *et al*. reported a meso-/microporous carbon with high percentage of mesopores of 52% through a dual-activation strategy^12^. In addition to creating mesopores directly, introducing mesoporous materials such as graphene and carbon nanotube as substrates for PCs could also construct rational structures^13^. Those materials showed enhanced electrochemical properties thanks to their hierarchical structures. Besides, tuning physiochemical and electronic properties of PCs by heteroatomic doping is a promising approach to fulfilling the requirements for high-performance supercapacitors^14,15^. Through introducing defects into the lattice and altering the electron distribution, heteroatoms such as oxygen (O), fluorine (F), nitrogen (N), sulfur (S), phosphorus (P) and boron (B) could provide sites for specific adsorption and improve wetting property, which will further induce extra pseudo-capacitance and promote charge mobility in the doped PCs. In most cases, heteroatoms were introduced artificially by adding organic agents into carbon sources. For example, melamine and triphenylphosphine (TPP) are typical agents used for N and P doping, respectively. Although fabricating hierarchical structures and doping from chemical agents could provide materials with enhancing properties, achieving those goals in more cost-effective ways is of great importance as far as practical application is concerned.

So far, a variety of precursors such as fossil materials, polymers and waste biomaterials have been applied to produce ACs^16–20^. Among them, waste biomaterials have attracted strong interests thanks to their renewability, readily availability, economic and environmental friendliness^21,22^. For instance, the PC prepared via activation of shiitake mushroom shows high specific capacitances in a three-electrode system^23^. The PC microflakes originating from willow catkins deliver specific capacitance of 233.1 F·g^−1^ at 1 A·g^−1^ and exhibit 82.9% capacitance retention at 20 A·g^−1^ in 2 M KOH^24^. Therefore, exploring high-performance PCs from the nature is of great promise.

Herein, we report a P, O co-doped carbon with meso-/microporous structures derived from cicada slough, a widely distributed biomass-waste in summer. After activation, cicada sloughs, rich in amino acid, protein, chitin, P and some microelements, converted into a surface functionalized meso-/microporous carbon. It shows a high SSA of 1945 m^2^·g^−1^ and a O, P co-doping amount of more than 18 wt.%. Thanks to its rational porous structure and heteroatoms doping, when tested as electrode material for supercapacitors, the as-prepared PC presents higher specific capacitance, excellent rate and cycling performance in both acid and alkaline solutions.

## Results

**Figure 1:**
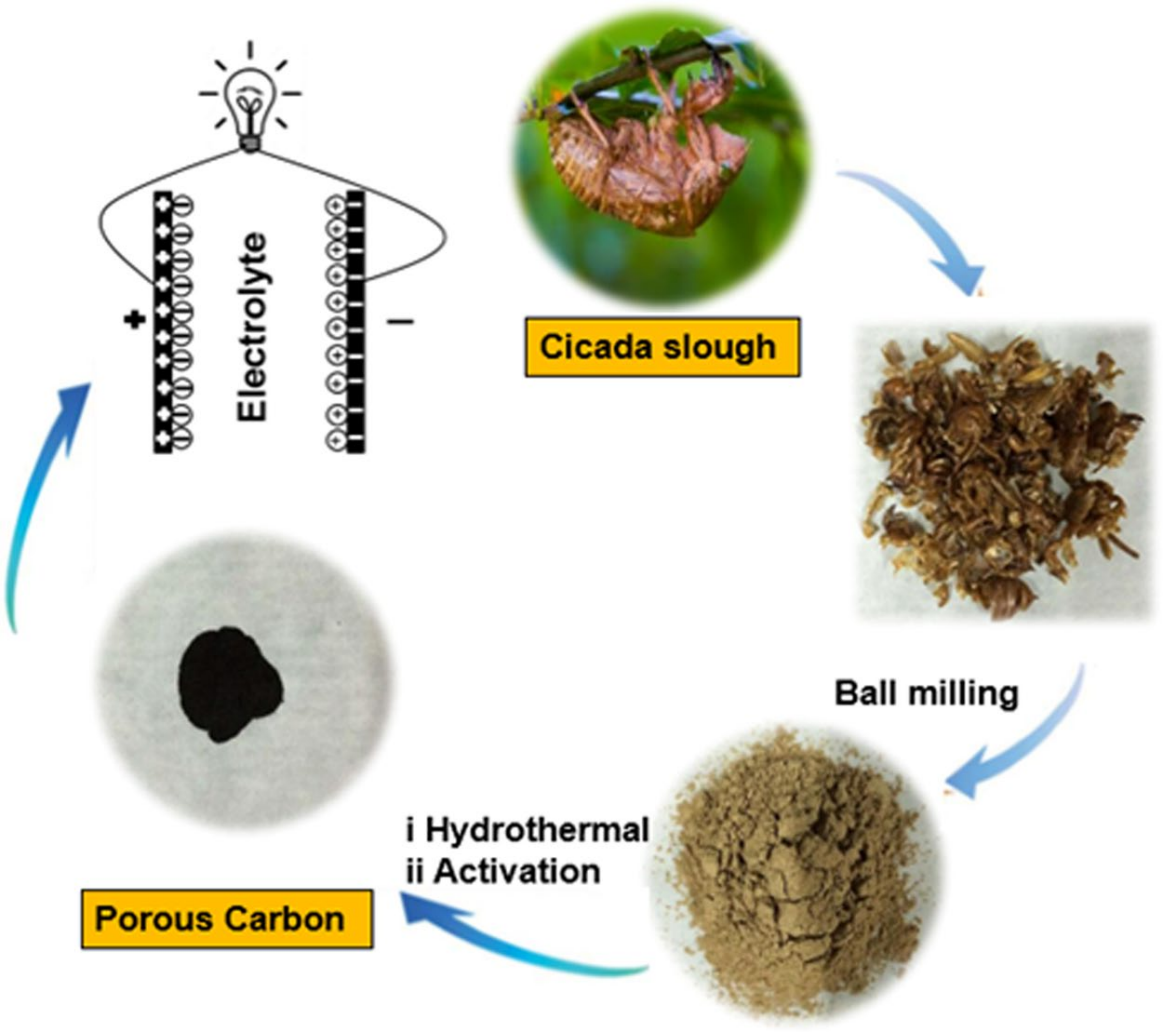
Illustration of the preparation process of the PC.

The preparation of the cicada sloughs-based PC is illustrated in Fig. 1. The approaches to converting ball-milled cicada sloughs into PC include hydrothermal treatment and the followed pyrolysis taking H_3_PO_4_ as the activation agent. For comparison, the hydrothermal product was pyrolyzed directly without H_3_PO_4_ and named PC_untreated_. The details are shown in the Methods section.

**Figure 2:**
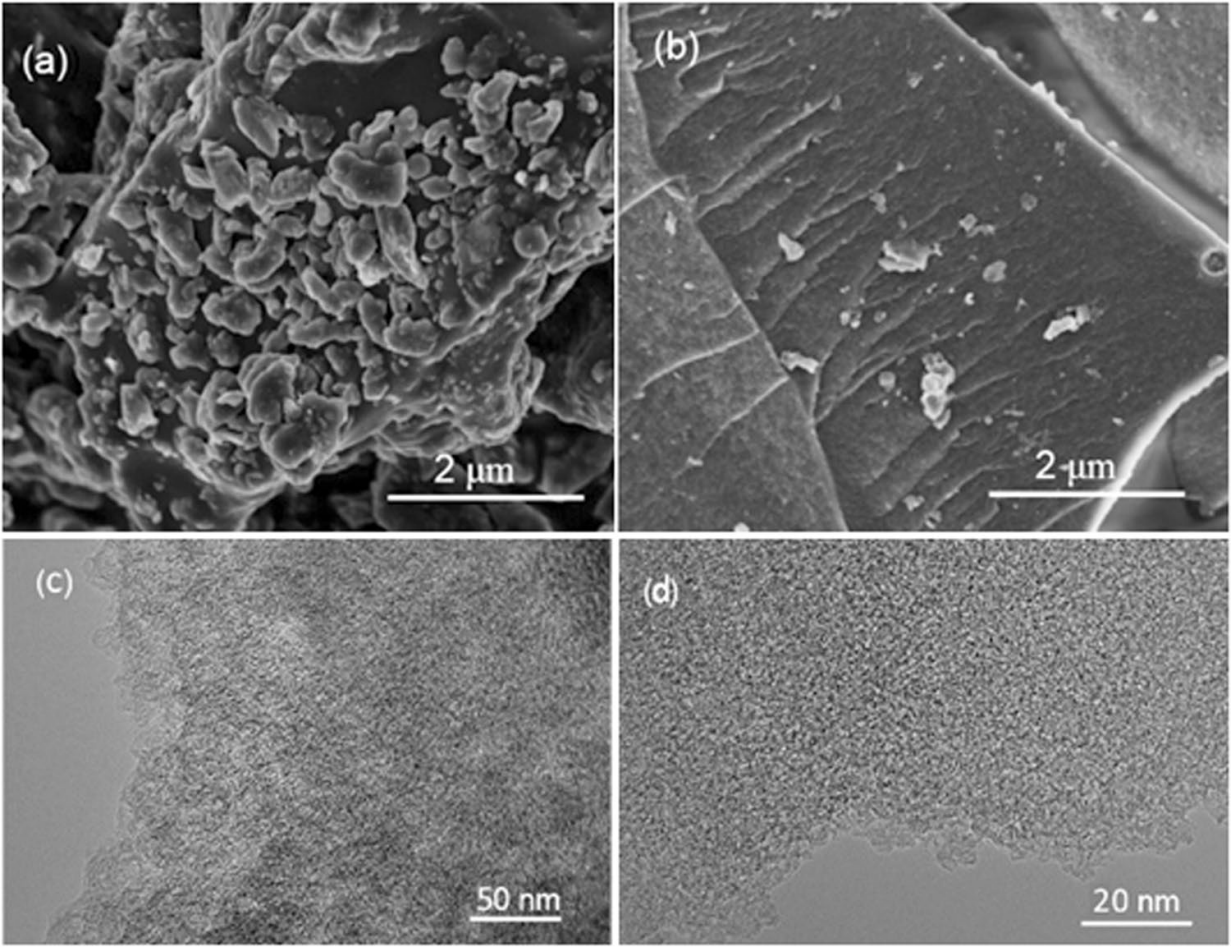
SEM images of (**a**) PC_untreated_ and (**b**) PC, and (**c**,**d**) TEM images of PC.

As shown in Fig. 2a, direct pyrolysis produced irregular carbon particles attached with lots of scattered small particles. However, those small particles were removed in the existence of H_3_PO_4_, exposing rough surfaces caused by the formation of large amounts of pores (Fig. 2b). Besides, according to the EDX results, some elements including Al, Mg, Ca and Si existed in PC_untreated_ and disappeared in PC, revealing that H_3_PO_4_ not only acted as pore creator but also scavenger for impure components.

The TEM image (Fig. 2c) reveals that meso/microporous channels have been created successfully through activation. The uniform distributed meso/microporous structure of PC is beneficial to enhancing its electrochemical performances. Since the micropores can provide abundant sites to store charges and the inter-knit mesopores can facilitate ion transportation, the diffusion distance from mesopores to micropores could be shortened, enabling improved capacitance and fast response^25^. The disordered graphene-type structures observed at the edge of the PC (Fig. 2d) could promote electron transfer and lower intrinsic resistance^16^.

**Figure 3:**
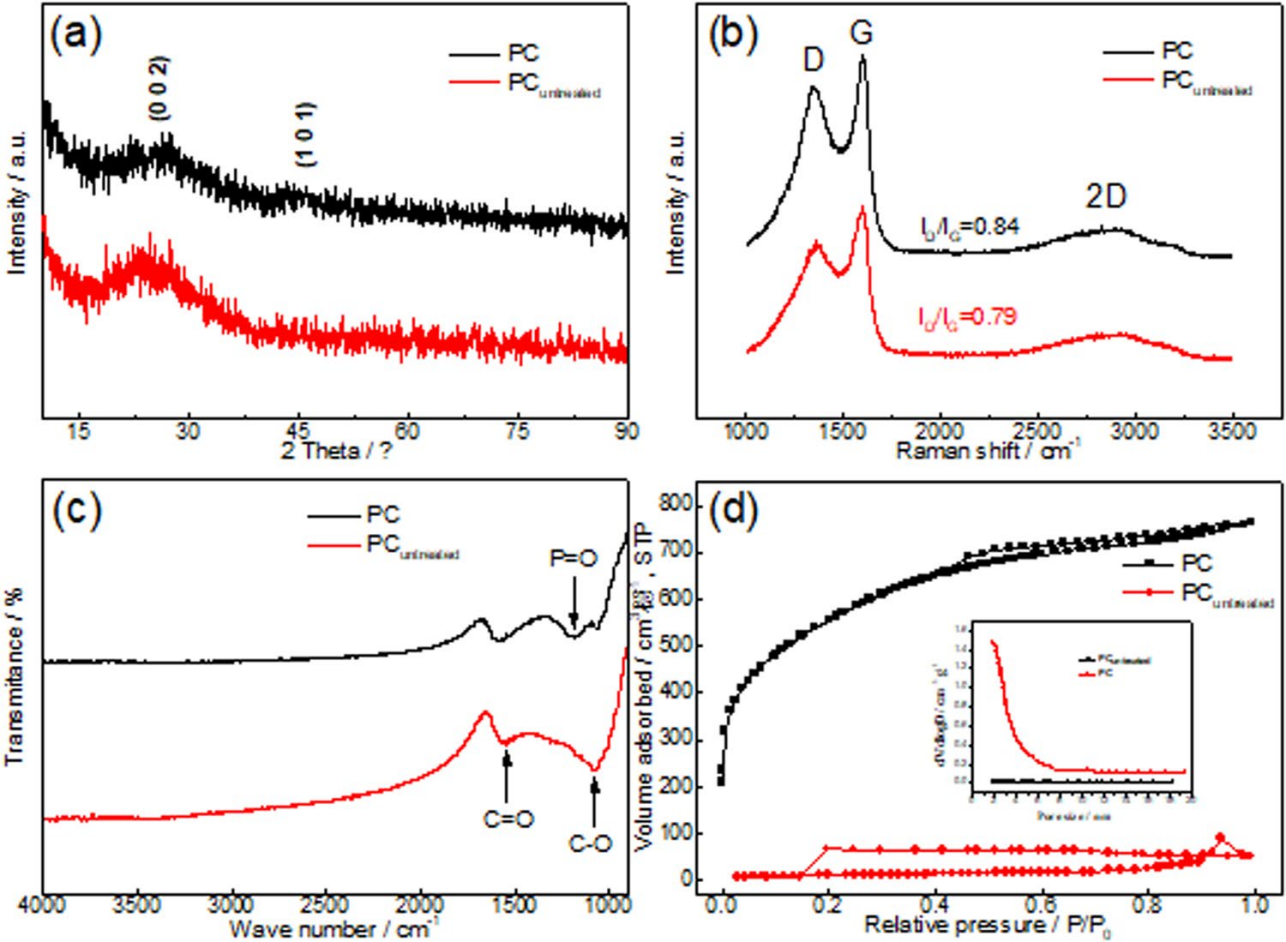
(**a**) XRD patterns, (**b**) Raman spectra, (**c**) FTIR spectra and (**d**) N_2_ adsorption-desorption results of PC and PC_untreated_.

Two broad peaks centered at 2θ = 25 and 46° in both XRD patterns of the PC and PC_untreated_ (Fig. 3a), corresponding to the (002) and (101) planes of graphite, respectively, indicate their low crystallinities^26^. As shown in Fig. 3b, both of their Raman spectra exhibit two specific peaks at 1365 (D band) and 1600 cm^−1^ (G band), relating to disordered carbon and sp^2^-bonded carbons, respectively^27^. The increased relative intensity of D and G bands (I_D_/I_G_) in the PC (0.84) implies that the H_3_PO_4_ activation could not only open closed pores but also etch graphitized carbon to create more disordered structures^28^.

Besides, with the help of H_3_PO_4_, more oxygen-containing functional groups were formed in the PC sample (Fig. 3c). For the PC_untreated_ sample, as shown in its FTIR spectra, two peaks at 1100 and 1630 cm^−1^ could be attributed to C-O and C=O stretches. Although it contains similar amount of phosphorus (4.87 wt.%) to that in the PC sample (4.58 wt.%), no peak belonging to C-P-O linkage is observed. However, for the PC sample, its FTIR spectra show a new peak at 1180 cm^−1^, which could be assigned to the stretching vibration of P=O groups^29^. In some formerly reported papers, H_3_PO_4_ activation could introduce P to ACs, but usually on a smaller level^11^. Therefore, in this case, the P-heteroatoms in P=O groups might come from both the H_3_PO_4_ and the existed P in carbon matrix, C-P=O^30^. Since the electronegativity of P (2.19) is lower than that of C (2.55), the existed P-heteroatoms are inclined to be functionalized by acid-oxidizing, a common way of getting surface functionalized carbons. Because of the inherently hydrophobic nature of carbon matrix, ion transportation inside the microporous structures is always unsatisfying. Those oxygen-containing functional groups on the material surfaces could improve their wettability, promote ion transfer and provide extra pseudo-capacitance^27^.

According to the N_2_ adsorption-desorption isotherms (Fig. 3d), the PC exhibits combined I/IV types adsorption-desorption isotherms (according to the IUPAC classification) with an apparent hysteresis loop at a wide relative pressure between 0.4 and 1.0, indicating the coexistence of micropores and mesopores. Also, thanks to H_3_PO_4_ activation creating large amounts of new pores, the PC presents higher Brunauer-Emmett-Teller (BET) surface (*S*_BET_) (1945 m^2^·g^−1^) and larger total pore volume (*V*_tot_) (1.184 cm^3^·g^−1^) than those of the PC_untreated_ (35.5 m^2^·g^−1^ and 0.07 cm^3^·g^−1^, respectively). The high *S*_BET_ and *V*_tot_ are beneficial to the electrochemical performance since they can ensure more ions adsorption sites and more interconnected ion diffusion pathways that favor ion transport into/from the PC electrode material^31^. According to their Barrett–Joyner–Halenda (BJH) pore size distribution plots (the inset of Fig. 3d), after the activation, the average pore diameter (*D*_av_) decreases from 43.9 nm (PC_untreated_) to 2.81 nm, suggesting that the activating agent creates a great deal of micropores and mesopores.

**Figure 4:**
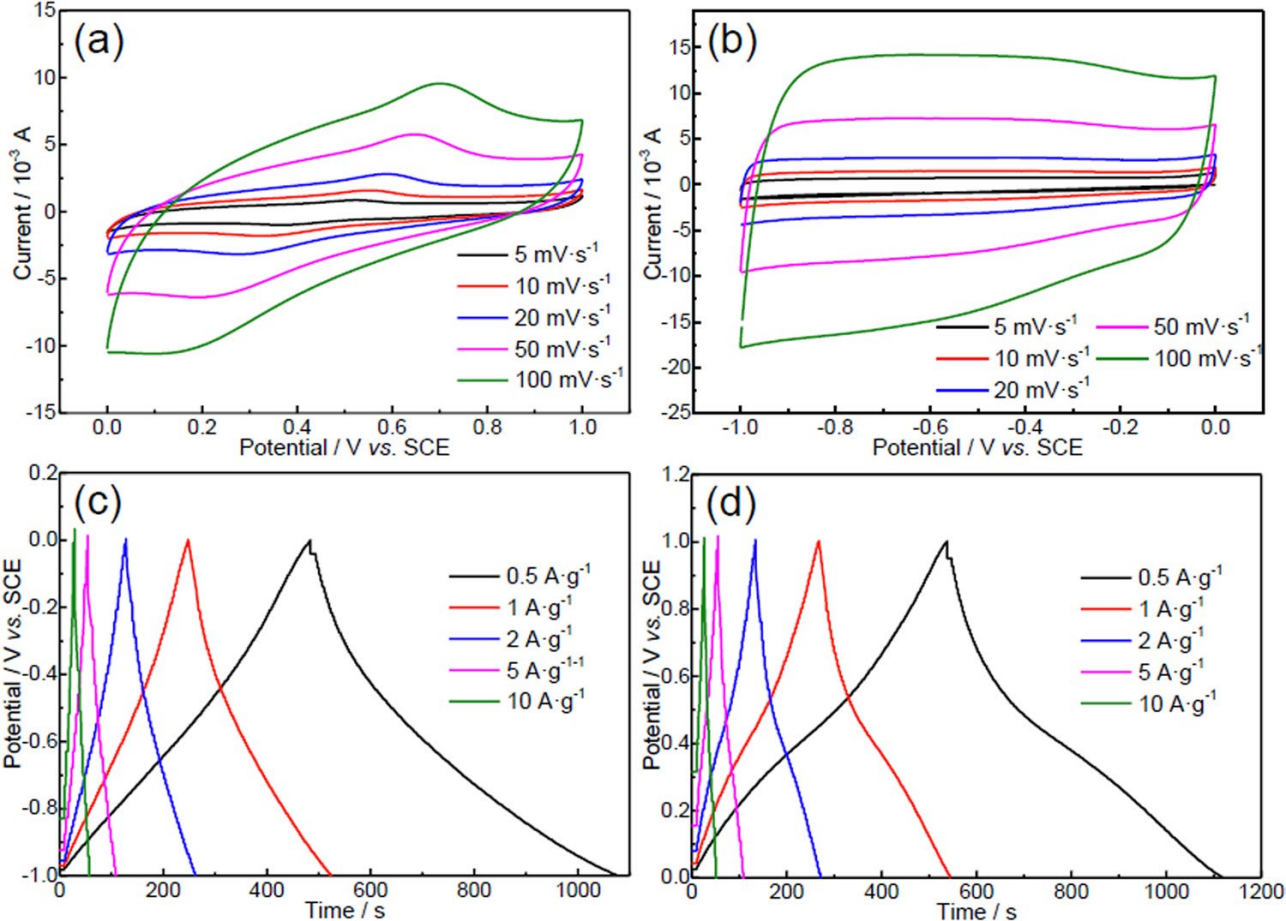
CV curves of the PC in (**a**) 1 M H_2_SO_4_ and (**b**) 6 M KOH, and its charge-discharge curves in (**c**) 1 M H_2_SO_4_ and (**d**) 6 M KOH.

As shown in Fig. 4a, the CV curves of the PC electrode in 1 M H_2_SO_4_ show a pair of peaks corresponding to the emergence of pseudo-capacitance (Fig. 4a), indicating that those surface functionalized groups, acting as favorable binding sites, significantly enhance its binding affinity toward H^+^. Also, a pair of broad peaks are observed in its CV curves when tested in alkaline solution (6 M KOH), which might be attributed to the specific adsorption of OH^−^ on C-P=O sites (Fig. 4b). Thanks to the functionalized groups and meso-/microporous structure of the PC, its CV curves still retain regular shapes at high potential scan rates, revealing its good rate performances in both acid and alkaline solutions. The symmetric charge-discharge curves at various current densities also indicate its good rate performance and electrochemical reversibility in those solutions (Fig. 4c,d).

**Figure 5:**
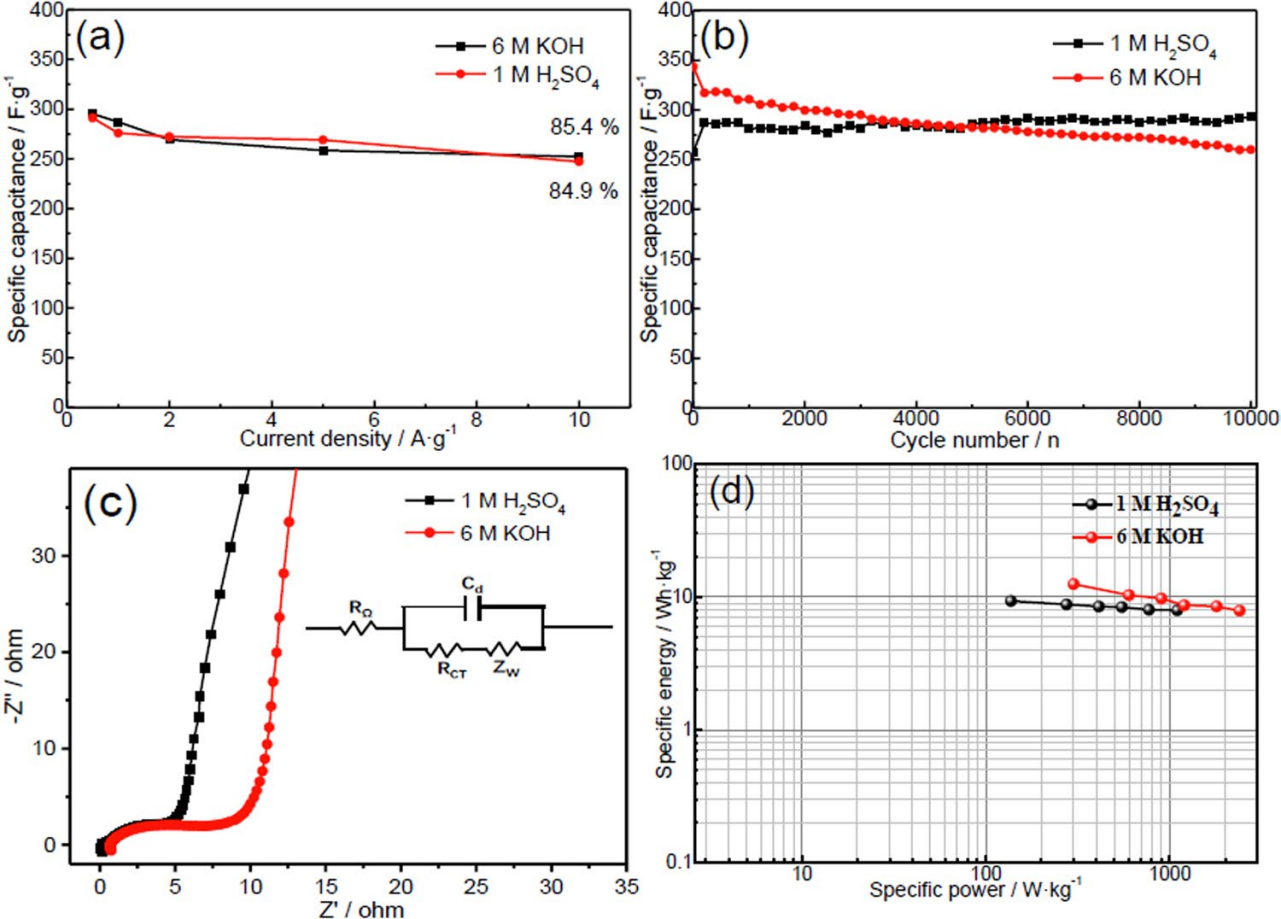
(**a**) Rate performance, (**b**) cycling stability and (**c**) Nyquist plots of the PC in acid and alkaline solutions; (**d**) Ragone plots of symmetric supercapacitors in 1 M H_2_SO_4_ and 6 M KOH (inset is equivalent electric circuit).

The specific capacitance was calculated from the discharge curves according to the following equation:

1 $${C}_{s}=\int \frac{Idt}{mdV}$$

where *C*_*s*_ is the specific capacitance (F·g^−1^), *I* is the discharge current (A), *dt* is the differential of discharge time, *m* is the mass of the active materials (g) and *dV* (V) is the differential of potential. As shown in Fig. 5a, the PC could deliver specific capacitances of 295.3 and 291.2 F·g^−1^ at 0.5 A·g^−1^ in 6 M KOH and 1 M H_2_SO_4_ electrolytes, respectively, which are superior to those of many biomaterial-derived carbons (Table S1)^29,32–40^. Although ion diffusing into the inner micropores is limited by the short time at high current density, its specific capacitances still maintain at 252 (6 M KOH) and 247 F·g^−1^ (1 M H_2_SO_4_) at 10 A·g^−1^, with capacitance retention of 85.4% and 84.9%, respectively. Its high capacitance and good rate stability can be attributed to the following factors: (1) the ultrahigh SSA and pore volume accommodating ions; (2) the well-connected meso-/microporous network enabling the thorough utilization of surface and fast ion transportation; and (3) the surface functionalized groups epsecially the doping of O and P inducing pseudo-capacitance and enhanced wettability^30,41^.

The cycling performance of the PC was conducted at the current density of 1 A·g^−1^ (Fig. 5b). In H_2_SO_4_ solution, the capacitance of PC increases during the initial 200 cycles because the H^+^ penetrating from the surface to the near-surface bodies could contribute to extra pseudo-capacitance^41^. However, in KOH solution, since OH^−^ could not infiltrate into the bodies easily because of its large size and its same electrical property with that of oxygen-containing groups repulsing each other, no activation process is observed. After 10000 cycles in H_2_SO_4_ solution, its capacitance remains at about 287 F·g^−1^ with no capacitance decay. In contrast, it falls 24% over 10000 cycles in KOH solution. This phenomenon is resulted from the pseudo-capacitance contribution of oxygen-containing functional groups, which is smaller in alkaline solution than that in acid solution, and those functional groups are not very stable in alkaline solutions^42^. If there is no functional groups, the AC will present stable cycling.

The differences of its electrochemical performances in acid and alkaline solutions could also be drawn from its EIS results in those solutions (Fig. 5c). The overall resistance of an electrode (resistance at the “knee” point, *R*_*knee*_) can be determined by the extrapolation of the capacitive line to the 45 ° line. The relationship between various resistances follows the equations below.

2 $${R}_{knee}={R}_{\Omega }+{R}_{P}+{R}_{D}$$

3 $${R}_{ct}={R}_{P}+{R}_{D}$$

The internal resistance of an electrode (notated as *R*_*Ω*_) could be obtained from the point intersecting with real axis at high frequency. The polarization resistance (*R*_*P*_), i.e. the diameter of semicircle, reveals penetrating ability of electrolyte into porous electrode. The diffusional resistance (*R*_*D*_), i.e. the length of 45 ° line in middle frequency region, indicates migration rate of ions from electrolyte inside pores to the surface of electrode. The lower charge transfer resistance (*R*_*ct*_ ≈ 4.1 Ω) in H_2_SO_4_ solution reveals that H^+^ could transfer easily in O, P-codoped porous carbons thanks to its smaller size and its different electrical property with that of oxygen-containing groups inducing smooth ion transfer. Besides resistances, the straight line in low frequency region is an important measurement of capacitive behavior and a vertical line suggests ideal capacitive performance. From this perspective, the PC electrode demonstrates better capacitive behavior in KOH solution because the capacitance mostly draws from electrochemical double-layers.

To further investigate the practical applicability of the PC, symmetric supercapacitors based on the PC in 6 M KOH and 1 M H_2_SO_4_ were assembled. The energy and power densities of the symmetrical supercapacitor systems were calculated according to the following equations:

4 $${\rm{E}}=\frac{C\,\int {V}^{2}dV}{2}$$

5 $${\rm{P}}={\rm{E}}/{\rm{t}}$$

where *E* (Wh·kg^−1^) is the specific energy density, *P* (W·kg^−1^) is the specific power density, *C* (F·g^−1^) is the specific capacitance of the total symmetrical system, *V* is the potential (V), and *t* is the discharge time (s). On the basis of the weights of the two electrodes, the specific energy densities are 12.5 Wh·kg^−1^ at the power density of 300 W·kg^−1^ in 6 M KOH and 9.32 Wh·kg^−1^ at 137 W·kg^−1^ in 1 M H_2_SO_4_ (Fig. 5d), which are superior to those of previously reported biomass-derived carbon materials, such as rice husk-derived PC (8.36 Wh·kg^−1^)^43^, a PC prepared by chicken feather (4.77 Wh·kg^−1^)^44^, and highly porous activated carbon electrodes from fibers of oil palm empty fruit bunches (4.297 Wh·kg^−1^)^45^. Those results demonstrate that this PC-based supercapacitor is of great promise for practical applications.

## Discussion

Just like harvesting energy from the sun, obtaining materials from the nature for practical applications is the thing that human beings have kept doing for thousands of years. Only when human learn and get inspired from the nature thoroughly could we innovate more incredible things. In the past years, fabricating hierarchical structure and heteroatom doping have been regarded as typical methods to improve the electrochemical properties of carbon-based materials. In this paper, we demonstrate that doping from the nature is an effective way of getting high-performance porous carbons^41^.

In summary, a porous carbon (PC) with high specific surface area, oxygen-containing groups and doped heteroatoms was prepared from cicada sloughs through pyrolysis and H_3_PO_4_ activation. The PC shows meso-/microporous structures with a high specific surface area (1945 m^2^·g^−1^) and a O, P co-doping ratio up to 18 wt.%. The doping effects of O and P on the capacitance is similar to those in carbon for lithium ion batteries, which lead to an increase of capacitance^41^. Of course, further detalied investigation should be investigated since different situation perhaps would result in different effects. Here, it exhibits excellent electrochemical performances, namely, high specific capacitances of 295 (6 M KOH) and 291 F·g^−1^ (1 M H_2_SO_4_) at 0.5 A·g^−1^, excellent cycling stability with no capacitance decay over 10000 cycles in H_2_SO_4_ solution, and good rate performance. In addition, the PC presents high energy density when assembled into symmetric supercapacitors, revealing its applicability in practical applications^46^. Therefore, on the basis of this study, further work could be focused on studying natural materials with abundant heteroatoms (P, N, O, S, and chlorides) and generating other valuable heteroatoms-doped carbons based on them.

## Methods

### Synthesis of the PC

The cleaned and dried cicada sloughs were ball-milled for 4 h at 600 rpm in a planet-type ball miller. The ball-milled cicada sloughs (1 g) were immersed in 35 mL 50 wt.% H_3_PO_4_ aqueous solution, stirred for a while to form homogeneous suspension and then transferred into a Teflon-lined stainless steel autoclave and heated at 180 °C for 12 h. The hydrothermal product was filtered (no washing) and dried at 80 °C. Next, the mixture was heated at 500 °C for 2 h in N_2_ atmosphere at a heating rate of 2 °C·min^−1^. Finally, the product was thoroughly washed with deionization water until the pH value reached to 7, and further dried at 80 °C for 12 h. For comparison, the ball-milled cicada sloughs without the H_3_PO_4_ treatment were pyrolyzed at the same condition, and the product was denoted as PC_untreated_.

### Characterization

The morphology and texture of the materials were characterized by field emission scanning electron microscopy (FESEM) on a Nova NanoSem 450 and field emission transmission electron microscopy (FETEM) on Tecnai G2 F20 S-Twin with acceleration voltage of 200 kV. Elemental compositions of the materials were measured with energy dispersive spectroscopy (EDS) microanalysis attached to the FESEM instrument. X-ray diffraction (XRD) was conducted on a D8 advance Bruker diffractometer with Cu K_α_ radiation (λ = 1.54056 Å). Raman spectra were recorded on Renishaw in Via Micro-Raman Spectroscopy. The surface functional groups of the materials were identified by Nicolet iS10 Fourier Transform Infrared (FTIR) Spectrometer using KBr pellets. Nitrogen adsorption-desorption isotherms were measured at 77 K on an AUTOSORB-IQ.

### Electrochemical measurements

The working electrodes consist of 80 wt.% active materials, 10 wt.% acetylene black and 10 wt.% polytetrafluoroethylene (PTFE) binder. Then the mixture was coated on a graphite rod (for 1 M H_2_SO_4_) or a nickel mesh (for 6 M KOH) with a mass loading density of 1 mg·cm^−2^, dried in vacuum at 80 °C for 12 h. In three electrodes systems, the graphite rob (for 1 M H_2_SO_4_) or nickel mesh (for 6 M KOH) and a saturated calomel electrode (SCE) were used as the counter and reference electrodes, respectively. Cyclic voltammetry (CV) and electrochemical impedance spectroscopy (EIS) were tested by a CHI400B electrochemical working station. The galvanostatic charge/discharge (GCD) and cycling stability tests were carried out on an automatic LAND battery test instrument^47,48^.

## Supplementary information

Supplementary Information
