# Supplementary material for: Oxygen/phosphorus co-doped porous carbon from cicada slough as high-performance electrode material for supercapacitors
Source: Sci Rep. 2019 Apr 1;9:5431. doi: 10.1038/s41598-019-41769-y (PMC6443656; doi:10.1038/s41598-019-41769-y)
Supplement: Supplementary file 1 — Supplementary Information [file 41598_2019_41769_MOESM1_ESM.pdf]

Supporting Information

Oxygen/phosphorus co-doped porous carbon from cicada slough as high-performance electrode material for supercapacitors

Bingwei Chen, [1,2] Wenzhuo Wu, [3] Chunyang Li, [1] Yanfang Wang, [1,2] Yi Zhang, [1,*] Lijun Fu, [1] Yusong Zhu, [1] Lixin Zhang, [2] and Yuping Wu[1,2,*]

[1] School of Energy Science and Engineering & Institute for Electrochemical Energy Storage, Nanjing Tech University, Nanjing 211816, China

[2] New Energy and Materials Laboratory (NEML), Department of Chemistry and Shanghai Key Laboratory of Molecular Catalysis and Innovative Materials, Fudan University, Shanghai 200433, China

[3] Guanghua Cambridge International School, Shanghai 201315, China

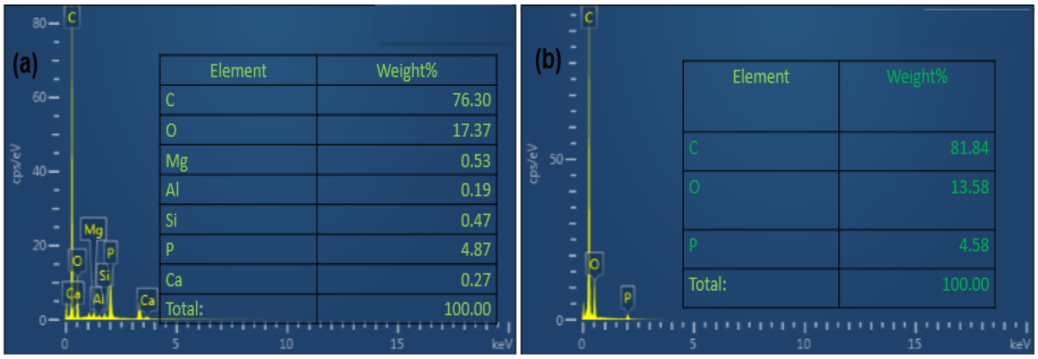

Element	Weight%
C	76.30
O	17.37
Mg	0.53
Al	0.19
Si	0.47
P	4.87
Ca	0.27
Total:	100.00

Element	Weight%
C	81.84
O	13.58
P	4.58
Total:	100.00

Figure S1. EDS spectra of (a) $PC_{untreated}$ and (b) PC.

[*] Email: wuyp@njtech.edu.cn; wuyp@fudan.edu.cn

Table S1. Comparison of the PC with other biomaterials-derived carbons

Material	Activating method	S_{BET} (m^2 g^{-1})	SC (F g^{-1})	Electrolyte	Ref.
shrimp shells	H_3PO_4	774	206	H_2SO_4	23
cotton	NH_3	778.6	245.3	H_2SO_4	26
tobacco stems	------	1749	167	Li_2SO_4	27
grape seed	HNO_3	1354	135	H_2SO_4	28
ginkgo shells	KOH	1775	178	KOH	29
rice husk	CO_2	1357	106	H_2SO_4	30
seed shell	KOH	1162	244	KOH	31
carrageenan	KOH	2502	230	KOH	32
seaweed	CO_2	1082	255	H_2SO_4	33
fungi	-----	80.08	196	KOH	34
cicada slough	H_3PO_4	1945	291	H_2SO_4	**this work**
cicada slough	H_3PO_4	1945	295	KOH	**this work**
